# Pregnancy outcomes in a malaria-exposed Malian cohort of women of child-bearing age

**DOI:** 10.3389/fmed.2022.1061538

**Published:** 2022-12-08

**Authors:** Santara Gaoussou, Oumar Attaher, Bruce Swihart, Moussa Traore, Soumaila Diarra, Ibrahim H. Soumbounou, Oulematou Ndiaye, Djibrilla Issiaka, Robert Morrison, Almahamoudou Mahamar, Patrick E. Duffy, Alassane Dicko, Michal Fried

**Affiliations:** ^1^Malaria Research and Training Center, University of Sciences, Techniques and Technologies of Bamako, Bamako, Mali; ^2^Biostatistics Research Branch, National Institute of Allergy and Infectious Diseases, National Institutes of Health, Bethesda, MD, United States; ^3^Laboratory of Malaria Immunology and Vaccinology, National Institute of Allergy and Infectious Diseases, National Institutes of Health, Bethesda, MD, United States

**Keywords:** pregnancy, miscarriage, preterm delivery, malaria, women of child-bearing age

## Abstract

In Sub-Saharan Africa, malaria continues to be associated with adverse pregnancy outcomes including stillbirth, early neonatal death, preterm delivery, and low birth weight. Current preventive measures are insufficient and new interventions are urgently needed. However, before such interventions can be tested in pregnant women, background information on pregnancy outcomes in this target population must be collected. We conducted an observational study in Ouélessébougou, Mali, a malaria-endemic area where first antenatal visit commonly occurs during the second trimester of pregnancy, hindering calculation of miscarriage rate in the population. To accurately determine the rate of miscarriage, 799 non-pregnant women of child-bearing age were enrolled and surveyed *via* monthly follow up visits that included pregnancy tests. Out of 505 women that completed the study, 364 became pregnant and 358 pregnancies were analyzed: 43 (12%) resulted in miscarriage, 28 (65.1%) occurred during the first trimester of pregnancy. We also determined rates of stillbirth, neonatal death, preterm delivery, and small for gestational age. The results showed high rate of miscarriage during the first trimester and established a basis to evaluate new interventions to prevent pregnancy malaria. This survey design enabled identification of first trimester miscarriages that are often missed by studies conducted in antenatal clinics.

**Clinical trial registration:**

[https://clinicaltrials.gov/], identifier [NCT0297 4608].

## Introduction

In Sub-Saharan Africa, malaria continues to be the primary cause of morbidity and mortality in young children and pregnant women. Adults develop immune responses that protect them from severe disease, but women become more susceptible to malaria during pregnancy, especially first pregnancy, that can result in adverse outcomes to the mother, the fetus/newborn or both (1). Adverse pregnancy outcomes related to pregnancy malaria include severe maternal anemia, preterm delivery (PTD), small for gestational age (SGA), low birthweight (LBW), stillbirth, and early neonatal death (2–5). These adverse outcomes are observed in both low and high malaria transmission areas (2, 3, 5).

To reduce malaria infection during pregnancy, WHO recommends that pregnant women receive preventive treatment with anti-malarial drug sulfadoxine-pyrimethamine (SP), termed intermittent preventive treatment during pregnancy (IPTp) at each scheduled antenatal care visit and at least 1 month apart starting from the second trimester. Although IPTp-SP has been recommended as part of standard care for pregnant women since 2000, WHO reported in 2021 that only 57% of pregnant women in Sub-Saharan Africa received at least 1 dose of IPTp-SP, and only 32% received 3 IPTp doses (4). Owing to safety concerns, IPTp-SP is contraindicated during the 1st trimester of pregnancy. In addition, the spread of SP-resistant parasites has been associated with worse pregnancy outcomes (6).

In this context, a vaccine to protect women from pregnancy malaria (PM) is urgently needed, and two types of vaccines are currently being investigated. The first vaccine type is based on naturally acquired immunity to PM. PM is caused by *P. falciparum*-infected erythrocytes that bind to the placental receptor chondroitin sulfate A (CSA) (7). Women become resistant to pregnancy malaria over successive pregnancies, as they acquire antibodies that target surface proteins of placental parasites and block parasite adhesion to CSA (8). This vaccine is based on a major infected erythrocyte surface protein (VAR2CSA) expressed by placental parasites and mediating their adhesion to CSA (8, 9). Two VAR2CSA-based products have been evaluated for safety in phase I clinical trials (10, 11). This vaccine can be administered to adolescent females prior to becoming pregnant. However, at this early stage of development, it is unknown whether a booster dose during pregnancy will be required.

The second vaccine type prevents human infection and is represented by PfSPZ Vaccine (Sanaria, Inc.), a whole organism vaccine product comprised of radiation-attenuated sporozoites (12). This vaccine is not specific for PM and is intended to prevent infection. In studies conducted in malaria-naïve individuals, this vaccine provided sterile immunity from challenge with a homologous and a heterologous strain of *P. falciparum* (12, 13). In a study conducted in Mali, 26% of adult vaccinees were protected from natural infection with heterologous strains during the following 6 months (14). Safety has been demonstrated in non-pregnant women aged 18–50 years (14) but no trials of PfSPZ Vaccine have enrolled pregnant women.

Until the recent FDA approval of Tdap vaccine for maternal immunization, no vaccine had ever been licensed for use in pregnant women. Further, the safety and efficacy of promising candidates against pregnancy malaria must be tested in this population. Because malaria infection itself is associated with poor pregnancy outcomes, background information on pregnancy outcomes in the target population is needed before initiating trials in pregnant women. Baseline rates of poor pregnancy outcomes will inform the interpretation of adverse outcomes during clinical trials, including their potential relationships to the intervention versus malaria infection or other risk factors.

To collect this background information, we established two cohorts of women residing in Ouélessébougou, Mali. In the first cohort that was previously reported, pregnant women enrolled during routine antenatal clinic visits (15), with the majority of women (70%) enrolled during their 2nd trimester of pregnancy. The most common adverse outcomes in that cohort included PTD and perinatal death occurring in 4.7 and 4.1% of the pregnancies, respectively. PTD and neonatal death were more common among primigravidae compared to multigravidae (15).

In Mali, similar to other sites in Sub-Saharan Africa (16), a majority of pregnant women make their first antenatal clinic visit during the second trimester (17), limiting our ability to collect information on miscarriage rates. This complicates the collection of accurate miscarriage rate data, because most miscarriages occur in first trimester, often before pregnancy is clinically recognized (18). The primary goal of the current study is to determine the miscarriage rate in all trimesters among women living in an area with high seasonal malaria transmission. To achieve this goal, we enrolled women of child-bearing age prior to becoming pregnant, then monitored them monthly with hCG testing until pregnancy diagnosis, and thereafter followed them to determine pregnancy outcome. This approach enabled an accurate and complete assessment of the miscarriage rate in the target population.

## Materials and methods

### Human subjects and clinical procedures

Women from Ouélessébougou, Mali enrolled in the study between November-December 2018 and follow-up was completed in October 2021. Women of child-bearing age (≥15 years) who were not using contraception nor breastfeeding for less than 12 months were invited to enroll. Exclusion criteria included temporary residence in the study area and conditions that could impair the ability of the woman to understand the study. The study protocol was approved by the Institutional Review Board of the National Institute of Allergy and Infectious Diseases, National Institutes of Health (ClinicalTrials.gov ID NCT02974608), and by the Ethics Committee of the Faculty of Medicine, Pharmacy and Dentistry at the University of Bamako, Mali. Written informed consent was obtained from study participants after receiving a study explanation form and oral explanation from the study clinicians in their native language. All experiments were performed in accordance with relevant guidelines and regulations.

Monthly pregnancy tests were conducted at the participant’s home. While enrolled in the study, women continued to receive their clinical care including routine antenatal care at their preferred public or private health center. Common preventive treatments provided at health centers included iron and folic acid supplementation as well as IPTp-SP. Malaria infections diagnosed during antenatal visits were treated with antimalarial drugs according to Mali Ministry of Health guidelines. No clinical laboratory tests such as malaria blood smear or hemoglobin levels were collected by the study. After a woman tested positive for pregnancy, hCG testing was repeated monthly through mid-2nd trimester to capture miscarriages during the first half of gestation. Home visits continued until the end of pregnancy to confirm women were still pregnant. Gestational age was determined by a trained obstetrician using ultrasound examination, which was performed in the 1st or 2nd trimester of pregnancy in 85% of women. Pregnancy outcomes, medical history and antenatal care information were collected 4–8 weeks after the end of pregnancy on case report forms, including information extracted from antenatal cards, as previously described (15).

### Pregnancy outcomes definitions

Miscarriage was defined as pregnancy ending before gestational week 28, stillbirth as a delivery of non-viable baby at a gestational age of ≥28 weeks (19), and neonatal death as death occurring in the first 4 weeks of life. Preterm delivery (PTD) was defined as birth before gestational age of 37 weeks. Small for gestational age (SGA) was defined as weight below the 10th percentile for gestational age according to INTERGROWTH-21 standards (20).

### Statistical analysis

Data were collected on standardized forms and scanned into the data base using DataFax (version 5.1.0, Clinical DataFax Systems, Inc., Hamilton, ON, Canada). Chi-squared test and Fisher’s exact test were used to compare proportions, and Mann–Whitney U test to compare continuous variables, between groups. Miscarriage rate by gestational week was calculated using previously described Life Table Analysis (18, 21). To evaluate the relation between risks factors and pregnancy outcomes, five data sets were created; each one had viable-term newborn as censored and one of the following as the observed event: miscarriage, stillbirth, neonatal death, PTD, and SGA. For each dataset, univariate and multivariate proportional hazard models were fitted using the survival package in R. Factors associated with adverse pregnancy outcomes were added to the models, including: gravidity; age group; miscarriage in the preceding pregnancy; malaria infection; number of IPTp doses, number of antenatal clinic visits; gestational age at 1st antenatal clinic visit, and a composite variable named “at least 1 known risk factor” that incorporated history of miscarriage, stillbirth, complicated delivery, height <150 cm, weight <45 kg and preeclampsia in the current pregnancy. Categorical covariates with fewer than 2 occurrences were excluded from the univariate model; for example, none of the 9 women whose pregnancy resulted in stillbirth had a reported malaria infection, and this predictor was not included in the model. Covariates with a *p*-value <0.1 in the univariate model were included in the multivariate model. The inclusion of age as a continuous variable was decided *a priori* for age-adjustment of the multivariate model.

## Results

### Study population

**FIGURE 1:**
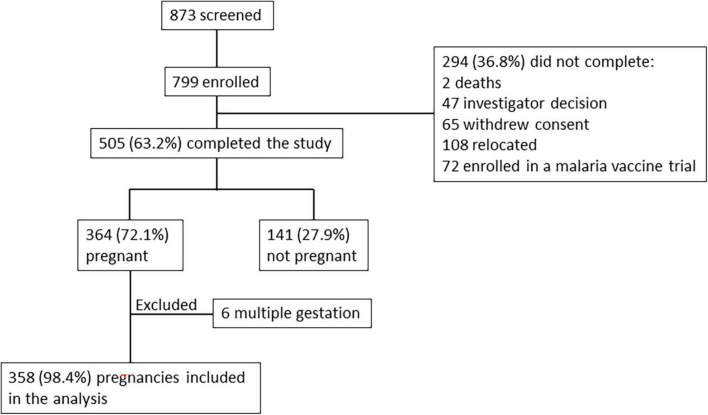
Flow chart of study population.

In total, 799 women of child-bearing age were enrolled into the study. After enrollment, monthly pregnancy tests were performed by the study team during follow-up visits at the participant homes. Of the 505 (63.2%) women who completed the study, 364 (72.1%) became pregnant, of which 6 were excluded from analysis due to multiple gestation (Figure 1). Median time from enrollment to pregnancy detection was 23.5 weeks (interquartile range 11.1–39.2 weeks), with no differences observed based on gravidity or age. Gestational age at the time of pregnancy detection was calculated based on ultrasound examination performed as early in pregnancy as possible. Pregnancy was detected at mean gestational age of 6 6/7 weeks (SD 1 week 6 days, range 3–12 weeks) and median gestational age of 6 6/7 weeks (interquartile range 5 6/7 weeks to 8 weeks). Although pregnancy tests were performed monthly, one missed visit was allowed resulting in 18 pregnancies that were detected between gestational week 10–12. The median time interval between births was 37.6 months.

Among women who became pregnant, 11.1% were primigravidae, and 19.5% were less than 20 years old (Table 1). Women who did not become pregnant during the study included 11.3% nulligravidae, 9.2% primigravidae, 9.9% secundigravidae, 29.1% multigravidae (2–5 previous pregnancies) and 40.4% grand multigravidae (≥6 previous pregnancies). Primigravidae attended their first antenatal clinical visit at a significantly earlier gestational age than other women, and grand multigravidae attended their first antenatal clinic visit at a later stage than primigravid, secundigravid and multigravid women (Table 2). Mean number of antenatal clinic visits was similar between primigravid and secundigravid women, and more frequent in both compared to multigravidae and grand multigravidae (Table 2).

**TABLE 1:** Study population (*n* = 358).

	*n* (%)
**Age**	
<20	70 (19.5)
20–35	242 (67.7)
>35	46 (12.8)
**Gravidity**	
Primigravid	40 (11.1)
Secundigravid	62 (17.3)
Multigravid	187 (52.4)
Grand multigravid	69 (19.2)
Number of ANC visits: mean (SD)	2.5 (1.4)
**SP-IPTp doses**	
0	53 (14.8)
1–2	200 (56)
≥3	105 (29.2)
Used ITN	343 (95.8)

**TABLE 2:** Antenatal clinic visits.

	Gestational age at 1st ANC		Antenatal clinic visits	
		
	Median (IQR)	*P*-value for comparison to gravid group	Mean (SD)	*P*-value for comparison to gravid group
Primigravid	18.1 (14.8–21.2)	Secundigravid, *p* = 0.02	3.3 (1.3)	Secundigravid, NS
		Multigravid, *p* < 0.0001		Multigravid, *p* = 0.02
		Grandmultigravid, *p* = 0.009		Grandmultigravid, *p* = 0.003
Secundigravid	20.7 (16.3–25.9)	Multigravid, NS	3.1 (1.3)	Multigravid, *p* = 0.048
		Grandmultigravid, *p* = 0.009		Grandmultigravid, *p* = 0.007
Multigravid	21.9 (18.5–25.8)	Grandmultigravid, *p* = 0.04	2.7 (1.1)	Grandmultigravid, NS
Grand multigravid	25.0 (20.3–28.4)		2.4 (1.0)	

IQR, interquartile range; NS, not significant.

The majority of pregnant women [*n* = 305 (85.2%)] received at least one dose of IPTp-SP, and 29.2% of pregnant women received 3 or more IPTp-SP doses, similar to reports from other countries published by the World Health Organization (4). The median time interval between IPTp-SP doses was 6 weeks (interquartile range 4.9 to 8.9 weeks). Nearly all women reported using insecticide-treated bed net (ITN) and received tetanus toxoid vaccine (97.2%).

Clinical laboratory studies such as complete blood count or malaria test were performed on pregnant women at antenatal clinics when clinically indicated. 34 (9.5%) women had a documented malaria infection diagnosed by rapid diagnostic test or blood smear microscopy. Hemoglobin levels of <11 gr/dl were reported in 13 (3.6%) women with one case of severe anemia. Two women (0.6%) presented with pre-eclampsia. Other risk factors for adverse pregnancy outcomes included history of complicated delivery [*n* = 12 (3.5%)], previous miscarriage or stillbirth [*n* = 76 (21.2%)], height < 150 cm [*n* = 4 (1.1%)], and weight < 45 kg [*n* = 5 (1.4%)].

### Pregnancy outcomes

**FIGURE 2:**
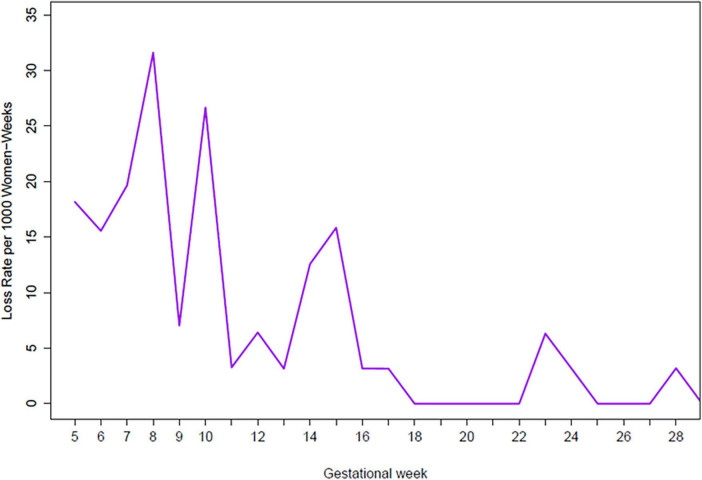
Miscarriage rate by gestational week.

The most common adverse pregnancy outcomes were small for gestational age (SGA), miscarriage and PTD (Table 3A). The majority of miscarriages (65.1%) occurred during the first trimester with highest weekly miscarriage rates of 32 and 27 miscarriages per 1,000 women-weeks observed at gestational weeks 8 and 10. From week 16, rates were below 10 miscarriages per 1,000 women-weeks (Figure 2 and Supplementary Table 1). The miscarriage rate was higher in women aged >35 years compared to women aged 20–35 years (Table 3C). PTD was more common among primigravidae versus multigravidae (Table 3B). SGA was more common among women aged <20 years (Table 3B). Percentages of stillbirth and neonatal death were similar between women of different gravidity or age groups (Table 3).

**TABLE 3A:** Adverse outcomes in the study population.

	*n*	% (95% CI)^1^
Miscarriage	43	12.0 (8.8–15.8)
Stillbirth	9	2.5 (1.2–4.7)
Neonatal death	9	2.5 (1.2–4.7)
PTD	17	4.8 (2.8–7.5)
SGA	49	13.7 (10.3–17.7)
LBW	12	3.4 (1.7–5.8)

^1^Percent of all pregnancies. PTD, preterm delivery; SGA, small for gestational age; LBW, low birth weight.

**TABLE 3B:** Adverse outcomes in the study population stratified by gravidity.

	Primigravid			Secundigravid			Multigravid		Grand multigravid		
				
	*n*	% (95% CI)	*P*-value^1^	*n*	% (95% CI)	*P*-value^1^	*n*	% (95% CI)	*n*	% (95% CI)	*P*-value^1^
Miscarriage	4	10 (2.8–23.7)	NS	4	6.5 (1.8–15.7)	NS	21	11.2 (7.1–16.6)	14	20.3 (11.6–31.7)	0.07
Stillbirth	0	0	NS	2	3.2 (0.4–11.2)	NS	5	2.7 (0.9–6.1)	2	2.9 (0.4–10.1)	NS
Neonatal death	1	2.5 (0.06–13.2)	NS	2	3.2 (0.4–11.2)	NS	3	1.6 (0.3–4.6)	3	4.3 (0.9–12.2)	NS
PTD	7	17.5 (7.3–32.8)	0.002	1	1.6 (0.04–8.7)	NS	6	3.2 (1.2–6.8)	3	4.3 (0.9–12.2)	NS
SGA	7	17.5 (7.3–32.8)	NS	11	17.7 (9.2–29.5)	NS	25	13.4 (8.8–19.1)	6	8.7 (3.3–18.0)	NS
LBW	6	15.0 (5.7–29.8)	0.04	2	3.2 (0.4–11.2)	NS	10	5.4 (2.6–9.6)	4	5.8 (1.6–14.2)	NS

^1^Fisher’s exact test for comparison to multigravida women. NS, not significant; PTD, preterm delivery; SGA, small for gestational age; LBW, low birth weight.

**TABLE 3C:** Adverse outcomes in the study population by age.

	Age <20			Age 20–35		Age >35		
			
	*n*	% (95% CI)	*P*-value^1^	*n*	% (95% CI)	*n*	% (95% CI)	*P*-value^1^
Miscarriage	6	8.6 (3.2–17.7)	NS	25	10.3 (6.8–12.8)	12	26.1 (14.3–41.1)	0.007
Stillbirth	1	1.4 (0.04–7.7)	NS	6	2.5 (0.9–5.3)	2	4.3 (0.5–14.8)	NS
Neonatal death	2	2.9 (0.4–9.9)	NS	5	2.1 (0.7–4.7)	2	4.3 (0.5–14.8)	NS
PTD	4	5.7 (1.6–14.0)	NS	11	4.5 (2.3–8.0)	2	4.3 (0.5–14.8)	NS
SGA	13	18.6 (10.3–29.7)	NS	33	13.6 (9.6–18.6)	3	6.5 (1.4–17.9)	NS
LBW	3	4.3 (0.9–12.0)	NS	17	7.0 (4.1–11.0)	2	4.4 (0.5–14.8)	NS

^1^Fisher’s exact test for comparison to women aged 20–35 years. NS, not significant; PTD, preterm delivery; SGA, small for gestational age; LBW, low birth weight.

Except for miscarriages, 94.3% deliveries occurred at a health facility setting (hospital or private clinic). Of the 43 miscarriages, 20 (46.5%) were admitted to the hospital. Miscarriages occurring at home were at earlier gestational age than those admitted to the hospital (median gestational age 10 3/7 weeks and 12 4/7 weeks, respectively) but the difference did not achieve statistical significance.

### Factors associated with adverse pregnancy outcomes

To identify factors associated with adverse outcomes, each of the following outcomes was evaluated in comparison to term live birth: miscarriage, stillbirth, neonatal death, PTD and SGA. For each outcome, univariate and multivariate proportional hazards models were fitted (Tables 4, 5). In addition to evaluating individual factors, a composite of multiple factors defined as risk factors by the antenatal clinic was evaluated. The composite named “at least 1 known risk factor” includes history of miscarriage, stillbirth and complicated delivery, height < 150 cm, weight < 45 kg and preeclampsia in the current pregnancy.

**TABLE 4:** Risks associated with fetal and neonatal death.

	Univariate		Multivariate	
		
Outcome	HR (95% CI)	*P*-value	HR (95% CI)	*P*-value
**Miscarriage**				
Age (continuous)	1.053 (1.007–1.101)	0.02		
**Age (group, years)**				
<20	0.862 (0.359–2.069)	0.7	0.755 (0.289–1.972)	0.6
20–35	Reference		Reference	
>35	2.659 (1.343–5.266)	0.005	2.932 (1.477–5.819)	0.002
**Gravidity**				
Primigravid	1.070 (0.384–2.986)	0.9		
Secundigravid	0.603 (0.206–1.766)	0.4		
Multigravid	Reference			
Grand multigravid	1.843 (0.943–3.604)	0.07		
At least 1 known risk factor	1.699 (0.905–3.190)	0.1		
Miscarriage last pregnancy	2.713 (1.091–6.745)	0.03	3.674 (1.378–9.797)	0.009
**Stillbirth**				
Age (continuous)	1.040 (0.953–1.136)	0.4	0.999 (0.897–1.113)	0.9
**Age (group)**				
<20	0.762 (0.093–6.280)	0.8		
20–35	Reference			
>35	1.891 (0.379–9.429)	0.4		
**Gravidity**				
Secundigravid	1.211 (0.268–5.482)	0.8		
Multigravid	Reference			
Grand multigravid	1.057 (0.200–5.583)	0.9		
IPTp-SP doses	0.271 (0.126–0.585)	0.0009	0.245 (0.110–0.546)	0.0006
Home delivery	3.732 (0.780–17.860)	0.1		
At least 1 known risk factor	3.502 (0.965–12.700)	0.06	4.088 (0.994–16.811)	0.05
ANC visits	0.770 (0.526–1.125)	0.2		
Gestational age at 1st ANC	0.937 (0.847–1.037)	0.2		
**Neonatal death**				
Age (continuous)	1.040 (0.946–1.144)	0.4	Model I: age and number of ANC visits 1.011 (0.909–1.23) Model II: age and number of IPTp doses 1.022 (0.926–1.129)	0.8 0.7
**Age (group)**				
<20	2.128 (0.421–10.750)	0.4		
20–35	Reference			
>35	2.156 (0.423–11.000)	0.4		
**Gravidity**				
Secundigravid	2.173 (0.366–12.910)	0.4		
Multigravid	Reference			
Grand multigravid	2.415 (0.517–11.290)	0.3		
IPTp-SP doses	0.467 (0.235–0.929)	0.03	Model II: age and number of IPTp doses 0.475 (0.226–0.999)	0.049
At least 1 known risk factor	0.805 (0.188–3.446)	0.8		
ANC visits	0.490 (0.276–0.868)	0.01	Model I: age and number of ANC visits 0.496 (0.265–0.928)	0.03
Gestational age at 1st ANC	1.027 (0.975–1.082)	0.3		

**TABLE 5:** Risks associated with preterm delivery and small for gestational age.

	Univariate		Multivariate	
		
Outcome	HR (95% CI)	*P*-value	HR (95% CI)	*P*-value
**PTD**				
Age (continuous)	0.948 (0.859–1.046)	0.3	0.972 (0.844–1.120)	0.7
**Age (group)**				
<20	1.314 (0.422–4.098)	0.6		
20–35	Reference			
>35	1.183 (0.259–5.404)	0.8		
**Gravidity**				
Primigravid	6.190 (2.111–18.148)	0.0009	4.197 (1.028–17.133)	0.04
Multigravid	Reference			
Grand multigravid	1.556 (0.387–6.254)	0.5	1.950 (0.251–15.147)	0.5
Malaria infection	3.312 (1.201–9.133)	0.02	2.710 (0.954–7.700)	0.06
IPTp-SP doses	0.912 (0.522–1.594)	0.7		
Home delivery	2.149 (0.493–9.371)	0.3		
At least 1 known risk factor	0.720 (0.205–2.531)	0.6		
ANC visits	0.793 (0.525–1.199)	0.3		
Gestational age at 1st ANC	0.941 (0.868–1.019)	0.1		
**SGA**				
Age (continuous)	0.951 (0.898–1.006)	0.08		
**Age (group)**				
<20	2.090 (1.114–3.922)	0.02		
20–35	Reference			
>35	0.721 (0.246–2.109)	0.6		
**Gravidity**				
Primigravid	1.834 (0.832–4.043)	0.1		
Secundigravid	1.450 (0.716–2.938)	0.3		
Multigravid	Reference			
Grand multigravid	0.707 (0.278–1.799)	0.5		
Malaria infection	0.707 (0.219–2.277)	0.6		
IPTp doses	0.870 (0.659–1.149)	0.3		
At least 1 known risk factor	0.731 (0.350–1.528)	0.4		
ANC visits	0.898 (0.737–1.094)	0.3		
Gestational age at 1st ANC	0.980 (0.943–1.018)	0.3		

Compared to women aged 20–35 years old, the risk of miscarriage was 2.7 times higher in women aged >35 years. History of miscarriage in the preceding pregnancy also increased the risk of miscarriage [Hazard Ratio (HR) 2.713 (95% CI: 1.091–6.745)]. In multivariate analysis, both age >35 years and miscarriage in the preceding pregnancy remained significant (Table 4).

Gravidity or age were not associated with increased risk of stillbirth. In univariate and multivariate analyses, the composite named “at least 1 known risk factor” was associated with increased HR of 3.5 and 4.1, respectively, while the number of IPTp-SP doses significantly reduced the risk for stillbirth (HR 0.3 and 0.2, respectively) (Table 4).

In univariate analysis, both the number of antenatal clinic visits and doses of IPTp-SP significantly reduced the risk of neonatal death. In multivariate analysis, the number of antenatal clinic visits and IPTp-SP doses were no longer significant, possibly due to high correlation between these covariates (*r* = 0.86, *p* = 0.02). Therefore, each of these factors was analyzed in separate multivariate models (models I and II). In multivariate models adjust for maternal age, number of antenatal clinic visits remained significant. Similarly, number of IPTp-SP doses remained significant after adjusting for maternal age (Table 4). The risk of PTD was 6.2-fold higher in primigravidae and 3.3-fold higher in women with reported malaria infection. In the multivariate model, both primigravidity and malaria infection during pregnancy increased the risk of PTD, but only gravidity achieved significance (Table 5). The risk of SGA was significantly higher in young women aged < 20 years (Table 5).

## Discussion

Malaria infection during pregnancy is associated with adverse outcomes. For example, we recently reported that malaria infection at our study site in Mali increased the risk of stillbirth and PTD in primigravidae, and early neonatal death in secundigravid and multigravid women (5). While pregnant women have historically been excluded from interventional clinical trials due to safety concerns, the need to test promising interventions in pregnant women has been increasingly recognized in recent years (22, 23). The primary objective of this surveillance study in an area with high seasonal malaria transmission was to determine the miscarriage rate as a baseline for future interventional trials.

A few studies have measured miscarriage rates during early pregnancies identified *via* daily measurement of urine HCG levels before clinical pregnancy [defined as pregnancy detected with conventional pregnancy testing or clinical examination (24)]. Based on two studies, early pregnancy loss accounted for 22% and 24.6% of pregnancies, whereas 11.6% and 7.9% of clinical pregnancies resulted in miscarriage (24, 25). Here, we report the rate of miscarriage in clinical pregnancies, which is relevant as a baseline miscarriage rate in interventional trials that use standard tools like pregnancy tests and LMP, where very early pregnancy losses may be missed. Of 358 pregnancies identified in this study, 43 (12%) resulted in miscarriage, which is within the range previously reported in other regions such as the United States and Europe (26, 27).

Consistent with previous studies (18, 28–30), the risk of miscarriage increased with age. In women aged >35 years, the instantaneous risk of miscarriage was 2.7 times higher compared to women aged 20–35 years. Risk of miscarriage has been reported to increase for women who miscarried in their previous pregnancy (29–31). This study supports those findings, as risk of miscarriage increased 3.7-fold in women that miscarried in their most recent pregnancy.

Although the primary goal of the study was to describe miscarriage rate, we are reporting other adverse outcomes that we documented in this cohort as well. In this survey, 4.8% of pregnancies resulted in PTD, similar to the rate we observed in the first cohort of pregnant women who we enrolled during antenatal clinic visits (15). Both gravidity and malaria infection increased the risk of PTD. The risk of PTD was 4.2-fold higher among primigravidae compared to multigravidae, and 2.7-fold higher in women with a history of malaria infection during pregnancy. This is consistent with our previous report of a longitudinal cohort study of pregnant women conducted in the same area, in which malaria infection in primigravidae was associated with increased risk of PTD (5).

We also observed that 9 (2.5%) pregnancies resulted in stillbirth and 9 (2.5%) in neonatal death. The rate of stillbirth is similar to earlier studies, that reported a stillbirth rate of 22.8 (19.9–24.8) per 1,000 births in Sub-Saharan Africa (32). A meta-analysis to evaluate risk factors associated with stillbirth found that malaria infection during pregnancy and at delivery increased the odds of stillbirth (3). In our previous longitudinal cohort study, we reported that malaria infection increased the risk of stillbirth for primigravidae and of early neonatal death for secundigravidae and multigravidae, while IPTp-SP significantly reduced these risks during the 3 weeks following drug administration (5).

This study was designed to collect information on baseline miscarriage rate and was not designed to evaluate the impact of malaria infection on risk of miscarriage or other adverse outcomes. Because malaria infection is often asymptomatic among adults in areas of stable transmission, frequent active testing for malaria infection and a large sample size would be required to confirm such associations. To date, one large population study of 17,613 women conducted in an area of low malaria transmission at the border of Thailand and Myanmar reported that both asymptomatic and symptomatic infection in the first trimester increased the odds of miscarriage (33). Relating malaria infection to miscarriage was possible because the women in that study attended weekly antenatal clinic for routine malaria tests starting in the first trimester (33, 34). At the site of the current study, women commonly attend their first antenatal clinic visit during the 2nd trimester (17), and 42 of the 43 miscarriages reported here occurred prior to the subjects’ 1st antenatal visit.

We speculate that small sample size limited our power to statistically confirm the association of malaria infection and PTD (*p* = 0.06) in the multivariate analysis, in addition to our study design that did not incorporate active testing for malaria infection. However, the increased rate of PTD in women with malaria infection is consistent with our previous report of a longitudinal cohort study of pregnant women conducted in the same area (5), and studies conducted in other areas. In a large study conducted in a low malaria transmission zone at the border between Thailand and Mynamar, infection with *P. falciparum* at gestational weeks 28–32 increased the odds of very early PTD, and infections after week 32 increased the odds of late PTD (2). In a cross-sectional study conducted in the Gambia, an area with seasonal malaria transmission, placental malaria significantly increased the odds of PTD (35). PTD is one of the major risk factors for neonatal death (36), thus, malaria control is one of the tools needed to reduce PTD and associated mortality.

As we previously observed, IPTp-SP significantly reduced risks of all adverse outcomes during the 3 weeks after drug administration. In this study, pregnant women received antenatal care at public or private health care facilities that generally limited malaria testing to occasions when clinically indicated. However, a large proportion of infections are asymptomatic (5) resulting in a limited number of women with a reported malaria infection episode. IPTp-SP can clear undiagnosed asymptomatic infections and suppress parasitemia while drug levels persist, thus reducing adverse outcomes associated with malaria infection (37). These benefits of IPTp, however, have limitations: SP is contraindicated in the first trimester; compliance with IPTp is generally low and a majority of women receive only 1–2 doses during pregnancy; SP has lost efficacy in East and Southern Africa due to the spread of drug-resistant parasites, and in these areas IPTp has been related to poorer pregnancy outcomes (6). Therefore, new tools are needed to control pregnancy malaria, and an effective and safe vaccine would be a valuable addition to our armamentarium.

In summary, preconception enrollment of women of child-bearing age enabled the identification of 1st trimester miscarriages. Overall, 43 (12%) pregnancies resulted in miscarriage, and 78 (21.8%) resulted in either miscarriage, stillbirth, neonatal death or preterm delivery. The main risks factors for miscarriage were age >35 years and a history of miscarriage in the preceding pregnancy, consistent with published data from other regions. Similar to our previous report from a longitudinal cohort study of pregnant women, primigravidity and malaria infection increased the risk of PTD. This background information will be useful in future vaccine trials as a baseline rate of poor pregnancy outcomes including miscarriage in the target population.

## Data availability statement

The original contributions presented in this study are included in this article/Supplementary material, further inquiries can be directed to the corresponding author.

## Ethics statement

The studies involving human participants were reviewed and approved by the National Institute of Allergy and Infectious Diseases at the US National Institutes of Health (ClinicalTrials.gov ID NCT02974608) and the Ethics Committee of the Faculty of Medicine, Pharmacy and Dentistry at the University of Bamako, Mali. The patients/participants provided their written informed consent to participate in this study.

## Author contributions

MF, AD, and PD designed the study. MF with contributions of PD and AD wrote the main text. BS and RM performed the statistical analysis. SG, OA, MT, SD, IS, ON, DI, and AM collected the data. All authors read and approved the final manuscript.
