# Supplementary material for: Pregnancy outcomes in a malaria-exposed Malian cohort of women of child-bearing age
Source: Front Med (Lausanne). 2022 Dec 8;9:1061538. doi: 10.3389/fmed.2022.1061538 (PMC9772013; doi:10.3389/fmed.2022.1061538)
Supplement: Supplementary file 1 [file Table_1.pdf]

Supplementary Table 1: number of fetuses in the study by gestational week

Gestational week	Number of fetuses	N miscarriages
Wk5	84	1
Wk6	176	2
Wk7	238	4
Wk8	280	8
Wk9	299	2
Wk10	311	8
Wk11	310	1
Wk12	317	2
Wk13	320	1
Wk14	321	4
Wk15	319	5
Wk16	318	1
Wk17	317	1
Wk18	317	0
Wk19	317	0
Wk20	318	0
Wk21	318	0
Wk22	318	0
Wk23	318	2
Wk24	316	1
Wk25	315	0
Wk26	315	0
Wk27	315	0
Wk28	314	1

Number of fetuses in the study: Cumulative number after subtracting those that left due to miscarriage in the previous weeks
